# Elevated concentration of beta2-microglobulin among patients with carpal tunnel syndrome in the course of primary Sjögren syndrome – a prospective observational study on 50 patients

**DOI:** 10.1007/s00296-024-05640-2

**Published:** 2024-07-06

**Authors:** Iga Kościńska-Shukla, Marta Jaskólska, Magdalena Chylińska, Dawid Jaskólski, Mariusz Siemiński, Michał Chmielewski

**Affiliations:** 1 Department of Rheumatology, Clinical Immunology, Geriatrics and Internal Medicine, Medical University of Gdańsk, Gdańsk, Poland; 2 Department of Adult Neurology, Medical University of Gdańsk, Gdańsk, Poland; 3 Department of Emergency Medicine, Medical University of Gdańsk, Gdańsk, Poland; 4 Second Clinic of Orthopaedics and Kinetic Organ Traumatology, Medical University of Gdańsk, Gdańsk, Poland

**Keywords:** Beta 2-microglobulin, Carpal tunnel syndrome, Amyloid neuropathy, Connective tissue diseases, Sjögren’s syndrome, Peripheral nervous system diseases

## Abstract

**Introduction:**

Sjögren’s syndrome (SS) is a chronic autoimmune disease characterized by lymphocytic infiltrates in the exocrine glands. Carpal tunnel syndrome (CTS) is suggested to be more frequent among SS patients than in the general population. The aim of this study was to seek associations between the CTS and the laboratory and clinical findings of SS patients.

**Methods:**

Fifty patients diagnosed with primary SS (pSS) were examined. Clinical evaluation by a rheumatologist and electrophysiological studies were conducted. Data on laboratory tests results was collected. Control group consisted of 50 sex and age-matched individuals with osteoarthritis (OA).

**Results:**

Out of 50 patients in the study group 27 (54%) were diagnosed with CTS. The prevalence of CTS among 50 individuals in the control group was 8%. Among pSS patients with CTS the joint involvement was not more common than in those from the non-CTS group [15 vs. 13 (*p* = 0.945)]. There was an expected difference in sleep disorders [18 vs. 9 (*p* = 0.012)] and paresthesia [23 vs. 13 (*p* = 0.024)]. The major finding was a significant difference in elevated beta2-microglobulin (B2MG) [23 vs. 13 (*p* = 0.024)]. Other studied factors, suggested in the literature as significant in the pSS-related neuropathy, were not statistically different between the groups.

**Conclusion:**

Our study confirms that CTS is more prevalent among pSS patients than in the general population and suggests that a new approach is required towards the pathogenesis of this phenomenon. We hypothesize that CTS is more associated with an overall disease activity than joint involvement as such.

## Introduction

The Sjögren syndrome (SS) was first described in the early 30s of the 20th century by an ophthalmologist Henrik Sjögren, who observed remarkable dryness of the eyes and oral cavity in a patient with articular involvement [1]. This connective tissue disease, characterized by lymphocytic infiltrates in the exocrine glands affects 0.2−1% of the general population, more commonly females (male-to-female ratio being 1:9) [2]. The symptoms usually develop in the fourth to sixth decade of life. Despite the dryness of the oral cavity and eyes being the most widely recognized symptoms, SS may lead to other, extraglandular manifestations, involving respiratory, cardiovascular, gastrointestinal, musculoskeletal and urinary systems [3, 4]. A common, yet understudied and underdiagnosed, manifestation of SS is the nervous system involvement, which affects both central (CNS) and peripheral nervous system (PNS) with the incidence of 6–48% and 2–60% respectively [5–8]. The PNS involvement may present itself in a variety of ways including sensory neuropathies, axonal sensorimotor polyneuropathy, mononeuropathy, multiple mononeuropathy, demyelinating polyradiculoneuropathy, cranial neuropathy, chronic inflammatory demyelinating polyneuropathy (CIPD), small fiber neuropathy and autonomic neuropathy [9, 10]. The pathophysiology behind the high incidence of PNS involvement is not yet known. The researchers refer to the potential importance of antinuclear antibodies (ANA), anti-SSA and anti-SSB antibodies and their affinity to different nervous fibers [11–13] as well as the role of rheumatoid factor (RF). It has been suggested that clinical features, such as longer course of the disease, history of vasculitis and Raynaud phenomenon could be the risk factors for development of PNS symptoms [14, 15]. Among the laboratory biomarkers factors such as lowered concentration of complement components, especially C3 and C4, hypergammaglobulinemia, vitamin D and vitamin B12 deficiencies are brought to attention [16–20].

The carpal tunnel is an anatomical region localized on the palmar area of the wrist. It is formed at the base by eight bones organized in two parallel rows (proximally scaphoid, lunate, triquetrum, and pisiform; distally trapezium, trapezoid, capitate, and hamate) and by a thick sheet of connective tissue (flexor retinaculum, otherwise known as transverse carpal ligament) at the top. Ten structures pass through the tunnel from the forearm to the hand - nine long flexor tendons (flexor pollicis longus, four tendons of flexor digitorum superficialis, and four tendons of flexor digitorum profundus) and the median nerve. A rigid surrounding, formed by bones and ligament, on one hand grants protection, on the other significantly limits the tolerance for an increase in the volume of any of the elements.

Carpal tunnel syndrome (CTS) is characterized by the presence of neurological signs and symptoms resulting from injury of the median nerve caused by compression within the carpal tunnel. Patients may experience paresthesia (tingling and numbness within the palm), sensory deficits, pain of the hand and forearm, weakness of the muscles of the hand. Neurologic signs and symptoms are accompanied by abnormalities in nerve conduction study of the median nerve [21].

Among patients diagnosed with rheumatic diseases, CTS is the most frequent among those with rheumatoid arthritis (RA) and systemic lupus erythematosus (SLE) [22]. This phenomenon is attributed in the literature to the joint abnormalities observed in the diseases – particularly with joint (including wrists) swelling in the periods of exacerbation [22], which leads to compression and consequently mechanical damage and ischemia of the median nerve. Until now, similar pathogenesis was suggested in the case of primary Sjögren Syndrome (pSS) due to commonly occurring arthritis [23]. However, our clinical observations do not support this hypothesis.

Based on the characteristics of CTS among pSS patients (frequently occurring bilaterally, poorly responding to local therapeutic procedures) we hypothesize that in fact CTS constitutes one of the presentations of PNS involvement. At the same time, it is common also among the general population, making it a good base for comparative analysis. The novelty and clinical significance of this study lies in the extensive data collected on the participants (laboratory findings, neurophysiological testing). The aim of this study is to determine potential risk factors, biomarkers and the pathomechanism of this peripheral neuropathy in patients diagnosed with pSS, allowing clinicians managing this group of patients in their daily practice to implement early diagnostic and therapeutic approaches.

## Methods

We studied a group of fifty consecutive patients with pSS from a large university-based hospital. All patients met the diagnostic criteria for pSS of the American European Consensus Group from 2002 [24] or the criteria of Sjögren’s International Collaborative Clinical Alliance from 2012 [25]. According to these, in case of absence of anti-SSA and/or anti-SSB with concomitant sicca symptoms, the patients underwent biopsy of labial salivary gland with evaluation of focus score, with a FS > = 1 (a cluster of > = 50 lymphocytes within a 4mm2 area) being indicative for the diagnosis. Patients with secondary SS were excluded from the study. Other exclusion criteria included: diagnosis of diabetes or other conditions leading to damage of nervous fibers, such as degenerative disc disease, abuse of alcohol or other toxic substances, organ failure (particularly liver and kidney), severe vitamin deficiencies with focus on the vitamin B complex and vitamin D as well as other neurological disorders and chronic renal failure. All patients underwent clinical evaluation by a rheumatologist and data on the medical history was collected. The control group consisted of age and sex-matched adults with diagnosed osteoarthritis who were under the care of the orthopedic outpatient clinic of the hospital. Patients were included in the study following an exclusion of any rheumatological diseases during evaluation by a certified rheumatologist.

All the patients underwent neurological assessment and nerve conduction studies of nine peripheral nerves (peroneal, tibial, sural, median bilaterally and ulnar unilaterally). Studies were performed under the protocol recommended by the American Association of Neuromuscular and Electrodiagnostic Medicine (AANEM) [26]. Medtronic’s Dantec Keypoint G4 equipment was used in a standardized environment (temperature of 32-34oC). To establish the lower limits of normal values 50 age-matched healthy individuals were examined. Polyneuropathies were classified following the European Standardized Telematic Tool to Evaluate Electrodiagnostic Methods (ESTEEM) guidelines. Classification included axonal, demyelinating, or mixed polyneuropathies [27]. Based on the results, coupled with physical examination (both performed and interpreted by a certified neurologist) the patients were divided into two groups – with peripheral nervous system involvement (PNS+) and without peripheral nervous system involvement (PNS-). Inclusion in the PNS + group involved finding of at least one of the following signs on the clinical examination: sensory deficit (tactile or vibration) or paresthesia or neuropathic pain found in at least one anatomical area innervated by specific peripheral nerves or neural roots; flaccid paresis found in any of the limbs; reduction of at least two tendon reflexes in at least one limb; signs of demyelination or axonal injury found in NCS of at least two nerves, behind typical localization of entrapments.

Moreover, for this analysis patients were divided into two groups based on the results of neurophysiological tests of median nerves – presenting signs of carpal tunnel syndrome (CTS) and free from carpal tunnel syndrome signs (non-CTS). Carpal tunnel syndrome was diagnosed in patients presenting typical signs and symptoms of CTS (any sign or symptom from the list: tingling and numbness within palm, pain of the hand and the forearm, sensory loss within the hand, loss of strength in hand) with positive Tinel’s sign or Phalen’s sign [28] accompanied by abnormal neural conduction in the corresponding median nerve.

The diagnostic techniques used in the nerve conduction studies of CTS have been described collectively by Werner and Andary [29]. All the tests performed in this study followed the AANEM recommendations [30]. According to these, the standard diagnostic approach to CTS includes the sensory conduction studies of the median nerve measured across the wrist and if abnormal compared with another sensory nerve of the symptomatic limb as well as additional studies in case of normal electrophysiological test result when conduction distance is greater than 8 cm. Moreover, it is suggested to perform a motor conduction studies from the thenar muscle and one other nerve in the symptomatic limb as well as optionally electromyography of muscles innervated by the C5 to Th1 spinal roots and comparison of motor nerve distal latencies of the median and ulnar nerves.

Disease activity and functional outcomes were assessed using the EULAR Sjögren’s Syndrome Disease Activity Index (ESSDAI) [31]. The scale focuses on 12 domains: cutaneous, respiratory, renal, articular, muscular, PNS, CNS, hematological, glandular, constitutional, lymphadenopathic, and biological. Depending on the score, each of the domains is divided into 3–4 levels of activity. The definition of moderately active disease involves the ESSDAI ≥ 5.

The subjective perception of the disease activity was assessed by the patients using EULAR Sjögren’s Syndrome Patient Reported Index (ESSPRI) [32]. This evaluation includes a questionnaire related to complaints such as: dryness, fatigue, and pain. Each of the components is measured with a 0–10 numerical scale, referring 0 as the lowest disease activity.

The study was approved by the Independent Bioethics Committee for Scientific Research of Medical University of Gdańsk, Poland (consent no. NKEBN/345/2011) and it met the ethical standards of the 1964 Helsinki declaration with successive amendments. All participants gave their written, informed consent.

The study was an observational study, performed on the results of patients under the care of our Department in the years 2012–2018.

### Statistical analysis

The normality of the distribution was checked using the Shapiro-Wilk test. Depending on its result, the continuous variables were compared using the Mann–Whitney U test or the Student T-test. Categorical variables were compared with the use of the Chi-squared test. A *p*-value of 0.05 or less was considered significant.

## Results

Our group consisted of 50 patients with a median age of 57.5 years (33–74). Of them 48 (96%) were female. The median age at pSS diagnosis was 53.5 years (23–69) with the median age of first symptoms of 46 years (18–66). The median disease duration since diagnosis in the group was 1.5 years, ranging from 1 to 15 years. The median time from the first symptoms to the diagnosis was 5 years (0–26). The median BMI of the participants was 25.1 (16.6–34.4).

Based on the neurophysiological examination 27 patients (54%) were classified into the CTS group and 23 into the non-CTS group. The prevalence of CTS among the control group was 8%, which corresponded with the epidemiologic data for the general population described in the literature.

There were no statistically significant differences between the groups in terms of age, BMI, age at the onset of symptoms, age at diagnosis, time elapsed from the first symptoms till the diagnosis or the disease duration. The detailed descriptives are presented in Table 1.

**Table 1:** Clinical characteristics of the CTS and the non-CTS patients’ groups

	CTS (*n* = 27)	Non-CTS (*n* = 23)	*p*
Age (years)	61	56	0,072
BMI (kg/m^2^)	26,17	23,14	0,350
Age at first symptoms (years)	49	44	0,173
Age at diagnosis (years)	57	50	0,084
Time till diagnosis (years)	7,7	6,65	0,799
Disease duration (years)	3	1	0,479

Among the laboratory findings, those involving the prevalence of antibodies, including SS specific anti-SSA and anti-SSB antibodies, rheumatoid factor (RF) and antineutrophil cytoplasmic antibodies (ANCA) did not differ between the groups. The classic meters of inflammation, such as elevated erythrocyte sedimentation rate (ESR), elevated concentrations of C-reactive protein (CRP) or fibrinogen were not significantly different between the groups. Decreased C3 and C4 component elements’ concentrations were observed in only 4 and 3 patients respectively in each of the analyzed groups. Prevalence of vitamin D and B12 deficiencies was similar in both groups, as were the abnormalities in the total blood count, such as leukopenia, neutropenia, lymphopenia, anemia. Moreover, in terms of clinical features, including the history of vasculitis and thyroid insufficiency or presence of Raynaud phenomenon there was no statistically significant difference between the groups. Interestingly, CTS was more prevalent among patients reporting symptoms suggesting peripheral nervous system (PNS) involvement (24 vs. 16; *p* = 0.089). The neurophysiological tests, however, excluding the cases of CTS, did not find signs of PNS involvement more commonly in that group of participants. Statistically significant differences were found between the groups in the following aspects: elevated B2MG concentration, defined as a concentration > 2.6 mg/l, which is the upper limit of normal in our laboratory, (23 vs. 13; *p* = 0.024), reporting paresthesia (23 vs. 13 patients; *p* = 0.024) and reporting sleep disorders (19 vs. 8; *p* = 0.012). As a standard proceeding, in all the patients with elevated B2MG concentrations a full diagnostic process was performed, including the electrophoresis of plasma proteins, immunoglobulin light chains assessment and testing for presence of a monoclonal protein. In justified cases a bone marrow biopsy was performed. In case of lymphoma suspicion the patients underwent ultrasonography of the peripheral lymph nodes and in case of doubts also a full-body CT scan for detection of pathological lymph nodes. Detailed descriptions of the group are included in Table 2 below.

**Table 2:** Prevalence of laboratory test abnormalities and clinical features in groups of CTS and non-CTS patients diagnosed with pSS

	non-CTS (*n* = 23)	CTS (*n* = 27)	*p*
POSITIVE SSA ANTIBODIES	15 (65.22%)	19 (70.37%)	0.542
POSITIVE SSB ANTIBODIES	12 (52.17%)	11 (40.74%)	0.346
POSITIVE ANCA	0 (0%)	0 (0%)	0.106
POSITIVE RF	14 (60.87%)	16 (59.26%)	0.969
DECREASED C3	4 (17.39%)	4 (14.81%)	0.821
DECREASED C4	3 (13.04%)	3 (11.11%)	0.639
HYPERGAMMAGLOBULINEMIA	12 (52.17%)	16 (59.26%)	0.774
ELEVATED ESR	7 (30.43%)	11 (40.74%)	0.449
ELEVATED CRP	3 (13.04%)	7 (25.93%)	0.256
**ELEVATED B2MG**	**13 (56.52%)**	**23 (85.19%)**	**0.024**
VITAMIN B12 DEFICIENCY	0 (0%)	3 (11.11%)	0.166
VITAMIN D DEFICIENCY	9 (39.13%)	15 (55.56%)	0.467
ELEVATED FIBRINOGEN	10 (43.48%)	16 (59.26%)	0.266
LEUKOPENIA	7 (30.43%)	11 (40.74%)	0.449
NEUTROPENIA	4 (17.39%)	8 (29.63%)	0.313
LYMPHOPENIA	5 (21.74%)	9 (33.33%)	0.363
ANEMIA	7 (30.43%)	12 (44.44%)	0.309
RAYNAUD PHENOMENON	7 (30.43%)	9 (33.33%)	0.546
VASCULITIS	5 (21.74%)	6 (22.22%)	0.967
JOINT INVOLVEMENT	13 (56.52%)	15 (55.56%)	0.945
HYPOTHYROIDISM	3 (13.04%)	3 (11.11%)	0.834
PNS INVOLVEMENT SUBJECTIVE	16 (69.57%)	24 (88.89%)	0.089
PNS INVOLVEMENT OBJECTIVE (EXCLUDING CTS)	9 (39.13%)	14 (51.85%)	0.368
**SLEEP DISORDERS**	**8 (34.78%)**	**19 (70.37%)**	**0.012**
**PARESTHESIA**	**13 (56.52%)**	**23 (85.19%)**	**0.024**

The patients underwent assessment in the direction of depressive disorders using Beck Depression Inventory [33]. Out of the whole group, 29 patients (58%) scored 10 or more points, suggestive of depressive episode, but no statistically significant difference was noted between the CTS and non-CTS groups (mean result 12.826 vs. 12.37 points; *p* = 0.639). A history of previous psychiatric treatment (mainly due to depression, anxiety and sleep disorders) was more prevalent among CTS group but without statistical significance (13 vs. 9; *p* = 0.522).

## Discussion

The present study demonstrates a significant positive correlation between the elevated B2MG and the presence of CTS in pSS patients. There were no statistically significant differences among other laboratory findings and CTS, including elevated ESR and CRP as well as clinical characteristics of the patients, including history of vasculitis, thyroid insufficiency and presence of Raynaud phenomenon.

The values of CRP and ESR are commonly used in clinical practice as disease activity measures. For instance, in the case of RA their role has been so well described, that their values constitute a part of a popular clinical tool for the measurement of disease activity – Disease Activity Score (DAS 28) [34]. However, according to the literature, CRP and ESR do not constitute trustworthy measures of the disease activity in SS. Similar to ESR and CRP, other parameters recognized as activity measures in rheumatic diseases, such as decreased C3 and C4 or increased fibrinogen concentration did not correlate with the occurrence of CTS in the studied group. Moreover, patients develop compounding decreases in concentrations of the morphotic elements as the disease progresses, making cytopenia one of the clinically utilized meters of disease activity. Cytopenia can develop in the course of SS or even precede typical sicca symptoms and delay the diagnosis [35]. Considering this aspect, we performed the analysis of cytopenia prevalence among the patients. No statistically significant difference between CTS and non-CTS patients was found.

Vasculitis is a group of several diagnostic entities that share the inflammation and consequently necrosis of the blood vessels of different diameters. A specific subtype, described as *vasculitic neuropathy* constitutes one of the reasons for nervous system dysfunction in rheumatic diseases. Inflammatory changes at the level of vasa nervorum impair the blood supply to the nervous fibres, leading to their damage, with time – irreversible [36, 37]. Small vessel disease is usually associated with ANCA antibodies [38–40], therefore their presence has been suggested in the literature as a potential biomarker of susceptibility to PNS involvement. Among our patients none of those in the CTS nor non-CTS group tested positive for ANCA antibodies.

Hypothyroidism is most frequently caused by chronic autoimmune thyroiditis (Hashimoto disease), however there are several more potential reasons for this disorder [41]. The severity of symptoms varies from mild impairment to disability. Although both muscular and neurological symptoms are well recognized complications of untreated thyroid hormone deficiency, their prevalence is not coherently described. Based on the literature, neurological manifestations (mostly sensorimotor neuropathy) are present in 10 to 70% of patients [42]. Additionally, increased BMI is suggested as an independent risk factor for CTS among hypothyroidism patients [43]. In the case of our patients none of these factors proved significant to the development of CTS.

Although some researchers considered the anti-SSA and anti-SSB antibodies potential culprits in the PNS involvement, there is plenty of discrepancy among the studies. Some of them highlight the role of anti-SSA antibodies [11], others attributing nearly equal importance to both anti-SSA and anti-SSB antibodies [13]. In the case of our group there was no statistically significant difference in the prevalence of either of the antibodies, neither in the CTS and non-CTS groups, nor in the population of patients diagnosed with PNS involvement other than the CTS. Literature reports suggest though, that patients seropositive for both of the antibodies present higher levels of B2MG [44]. Therefore, considering the association between B2MG concentration and prevalence of CTS there might be an indirect relationship between the antibody type and susceptibility to CTS.

Connective tissue disorders often co-occur with mental health problems, particularly depression. Symptoms such as persistent pain, joint deformities, pulmonary or cardiac manifestations substantially lower the quality of life of patients (QoL). Long-lasting, immunosuppressive treatment carries the risk of opportunistic infections and malignancies. Other aspects impairing daily functioning include among others: lack of support from family and friends, lack of understanding of patients’ complaints and symptoms, costs generated by healthcare visits and medication, feeling of insecurity related to the future (source of income, disease complications, side effects of the therapy). According to data published in 2021, 3% of the Polish population experienced at least one depressive episode [45]. Meanwhile, among our study group 29 patients (58%) scored 10 or more points in the Beck depression scale and 22 patients (44%) had a history of psychiatric treatment (mainly due to depression, anxiety and sleep disorders). Long-lasting, lowering the QoL symptoms of CTS, including sleep disorders and paresthesia, coupled with higher prevalence of psychiatric disorders in pSS population, may lead to the development of depression requiring pharmacological treatment.

### The role of beta2-microglobulin

B2MG is a naturally occurring polypeptide, encoded in chromosome 15, consisting of 100 amino acids, with a total molecular weight of 11 800 Da. Its binding with major histocompatibility complex II (MHC II) / human leukocyte antigen I (HLA I) is present on all nucleated cells [46]. Moreover, the complex created between the alpha chain of HLA I and B2MG is vital for antigen presentation [47, 48]. In healthy individuals, the majority of B2MG is excreted through renal filtration, maintaining a normal serum concentration at the level of 1–3 ug/ml [49]. Normal values may differ slightly depending on the laboratory. Elevated levels of B2MG are observed, among others, in myeloma, lymphoma, liver disease and renal failure. In case of renal failure, the B2MG concentrations are inversely correlated with the glomerular filtration rate and can reach levels even 60 times higher than normal [50, 51]. The significance of B2MG as a biomarker of other conditions has been overlooked for many years. Nowadays, however, there is evidence suggesting its importance in malignancies (including ovarian [52], prostate [53], breast [54], gallbladder [55] cancers and renal cell carcinoma [56]), systemic lupus erythematosus (SLE) and Still disease [57, 58] by corresponding B2MG concentrations with disease activity and survival. Based on literature, B2MG also plays a role in SS.

A big prospective study, conducted on 395 participants, showed that B2MG concentration, together with the free light chains of gamma-globulins best corresponded with the disease activity measured in ESSDAI [44]. In the case of our patients, hypergammaglobulinemia did not correlate with the presence of CTS diagnosis. However, the incidence of CTS correlated with the elevation of B2MG above 2.6 mg/l reaching a statistically significant difference, giving the basis to correlate the occurrence of CTS with the overall disease activity, rather than just the articular manifestation. Moreover, B2MG has a known potential for the formation of amyloid fibrils which tend to deposit in the osteoarticular system, leading among others to CTS symptoms. Such findings are well-described in end-stage kidney disease (ESKD) patients undergoing hemodialysis (HD) but the pathomechanism behind it has not been fully understood [59, 60].

The first reports of increased prevalence of CTS among ESKD come from 1975 paper by Warren and Otieno [61]. Initially this phenomenon was attributed to the kind of fistulas with coexisting upper limb oedema leading to compression of the median nerve. Histopathological findings of samples from CTS surgical specimens proved that theory wrong, revealing with Congo-red staining presence of amyloid [62]. Further investigations on synovial amyloid led to the discovery of B2MG [50]. The proof for systemic character of the B2MG deposition comes from autopsies of patients on chronic HD, specifically the rectal mucosa [63]. Currently CTS secondary to ESKD is a frequent clinical problem due to the increasing number of patients on chronic renal replacement therapy. However, the symptoms tend to appear later from the initiation of HD and are often more subtle due to improvement of dialysis technology, including an increased B2MG clearance [64].

In the case of ESKD patients the B2MG levels usually significantly exceed the normal range (as mentioned above reaching even 60-fold of the upper normal range). The concentrations observed in pSS do not reach such levels, unless in instances of concomitant hematological malignancy or kidney failure. Nevertheless, based on the literature there is no definite correlation between the serum levels of B2MG and risk of amyloidosis development [65]. Despite over 40 years of studies on the underlying cause of dialysis related amyloidosis (DRA) little is still understood. Researchers raise the matter of probable facilitating factors, but those have not been established yet. Therefore, some form of interaction between inflammatory proteins or antibodies occurring in pSS patients cannot be ruled out and requires further research in this area. A systemic involvement, like the one observed in DRA, might be a contributing factor to other extraglandular symptoms in pSS.

Our results allow us to suspect that the increased incidence of CTS in ESKD and SS patients may share a similar mechanism. To our knowledge, no such observation has been made until now. Undoubtedly, more research is required in this field, most probably involving histopathological examinations of samples obtained during surgical interventions of the carpal tunnel release. Should our hypothesis find its support in the results, it would constitute a substantial change in the diagnostic and therapeutic approach to SS patients with symptoms suggestive of CTS and raise the importance of immunosuppressive medication and local steroid administration above the traditional, orthopedic interventions.

## Limitations

The study was conducted in a tertiary care university hospital, potentially resulting in a bias in patient selection caused by referral.

Unequivocal contribution of neuropathy to the underlying systemic disease requires years of observation. Therefore, we are unable to state with all certainty that observed neuropathy does not result from other causes (except those identified as exclusion criteria). However, such distinction does not affect the presented results, significant for the essence of the study.

The main advantage of this study is a thorough neurological and rheumatological assessment performed by one certified specialist in each field. Much of the research in this topic and related topics was based on retrospective assessment. Additionally, only patients with pSS were included in the study, after excluding other significant diagnoses, which allows us to obtain the most precise information about a specific disease entity.

## Conclusion

The analysis of data on 50 pSS patients conducted by our team suggests the need for reassessment of CTS pathophysiology among SS patients. We hypothesize that CTS is in fact more of a presentation of PNS abnormalities found in SS patients rather than isolated mononeuropathy, which might affect the therapeutic approach to this group of patients. Moreover, based on hitherto clinical observations we hypothesize that beta2-microglobulin (B2MG) plays a significant role in the pathogenesis of CTS in SS patients.

Our results suggest the need of extended screening for CTS among patients diagnosed with SS and elevated B2MG concentration who report upper limb paresthesia. CTS, if untreated, leads to unpleasant symptoms, severely impairing the quality of life of our patients. Therefore, there is a substantial demand for more research into the matter, particularly at the molecular level, in order to find the most efficient therapeutic strategies for our patients.

## Data Availability

Parts of the data, regarding prevalence of peripheral nervous system involvement in primary Sjögren Syndrome patients, were presented during poster session on EULAR 2019 congress: Ann Rheum Dis, volume 78, supplement 2, year 2019, page A1173. 10.1136/annrheumdis-2019-eular.4851.
